# Connections Matter: Channeled Hydrogels to Improve Vascularization

**DOI:** 10.3389/fbioe.2014.00052

**Published:** 2014-11-14

**Authors:** Severin Muehleder, Aleksandr Ovsianikov, Johannes Zipperle, Heinz Redl, Wolfgang Holnthoner

**Affiliations:** ^1^Ludwig Boltzmann Institute for Experimental and Clinical Traumatology, AUVA Research Center, Vienna, Austria; ^2^Austrian Cluster for Tissue Regeneration, Vienna, Austria; ^3^Institute of Material Science and Technology, Vienna University of Technology, Vienna, Austria

**Keywords:** hydrogel, channel, tissue engineering, endothelial cells, vascularization

## Abstract

The use of cell-laden hydrogels to engineer soft tissue has been emerging within the past years. Despite, several newly developed and sophisticated techniques to encapsulate different cell types the importance of vascularization of the engineered constructs is often underestimated. As a result, cell death within a construct leads to impaired function and inclusion of the implant. Here, we discuss the fabrication of hollow channels within hydrogels as a promising strategy to facilitate vascularization. Furthermore, we present an overview on the feasible use of removable spacers, 3D laser-, and planar processing strategies to create channels within hydrogels. The implementation of these structures promotes control over cell distribution and increases oxygen transport and nutrient supply *in vitro*. However, many studies lack the use of endothelial cells in their approaches leaving out an important factor to enhance vessel ingrowth and anastomosis formation upon implantation. In addition, the adequate endothelial cell type needs to be considered to make these approaches bridge the gap to *in vivo* applications.

## Introduction

The blood and lymphatic vascular systems provide most tissues in our body with a supply of nutrients and oxygen and ensure proper waste removal. When cells grow beyond the diffusion range of oxygen, hypoxia initiates a sequence of events to release signals to attract new blood vessels. The same events are triggered when a cell-laden construct is implanted into a host. However, blood vessels require several weeks to invade a millimeter-sized implant for complete vascularization. As a result improper nutrient supply following cell death in the core of such an implant can cause problems in engineered tissue integration (Rouwkema et al., 2008). There are several vascularization strategies to address these issues including but not limited to cell-based approaches like co-culture and technological methods such as scaffold structures imitating blood vessels (Levenberg et al., 2005; Kirkpatrick et al., 2011; Novosel et al., 2011).

Currently, hydrogels have various applications in medicine such as contact lenses (Nicolson and Vogt, 2001), sealants in surgery (Fortelny et al., 2012) blood-contacting material or in wound healing, and esthetic surgery (Kirschner and Anseth, 2013). However, new approaches in tissue engineering and regenerative medicine are emerging since hydrogels can serve as scaffolds to protect and carry embedded cells or support drug delivery. Biocompatible hydrogels can be designed to mimic various extracellular environments by altering their molecular characteristics, shape and size, physical properties, and degradability (Seliktar, 2012). Additionally, they can promote cell signaling and differentiation through interactions with the designed matrix (Geckil et al., 2010). Still, vascularization of an implanted hydrogel is key to adequate implant inclusion. Inadequate blood supply can lead to inhomogeneous distribution and compromised differentiation of cells embedded in the implanted hydrogel. To optimize tissue integration and function, cell-containing hydrogels need to promote proper vascularization in order to ensure cell survival within the construct (Rouwkema et al., 2008). To address these issues scaffold design and microarchitecture need to be fine-tuned accordingly as they are known to be an important factor for blood vessel ingrowth independent of the cell type(s) embedded (Baiguera and Ribatti, 2013). Mainly, pore architecture, interconnectivity, and channel design can effectively facilitate vascularization of implanted hydrogels (Roy et al., 2003; Ko et al., 2007).

The aim of this review is to highlight and discuss efforts to design and produce interconnected channels within natural and synthetic hydrogels to generate vascularized constructs *in vitro* in order to facilitate cell survival and function. Furthermore, an updated overview on strategies to promote endothelial invasion and vascularization of channeled hydrogels is provided.

## Removable Structures

**Figure 1:**
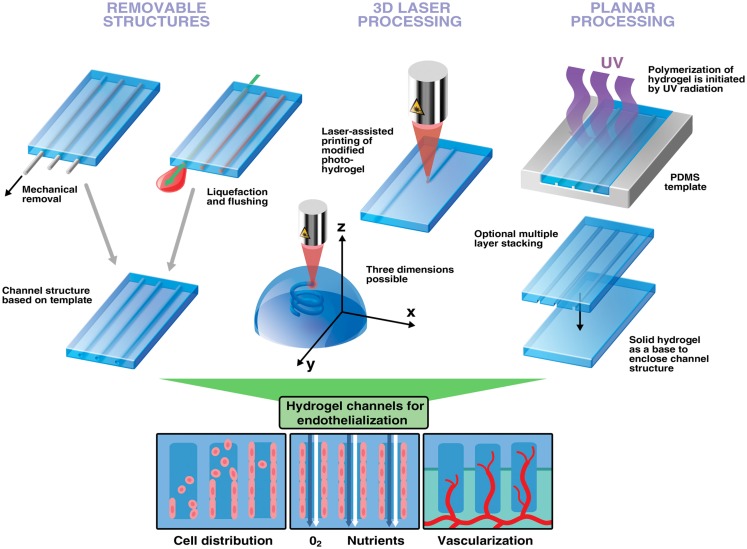
**Strategies to engineer channels and interconnected structures in hydrogels to improve vascularization**. Removable structures: hydrogels can be cast around non-sacrificial and sacrificial templates. Upon removal, defined channel structures are left behind. 3D laser processing can generate virtually any structures with a remarkable speed and spatial resolution. Depending on the material photo-reactive groups can be cleaved to initiate hydrogel network dissociation or activated for polymerization. Furthermore, photocoupling can be used to functionalize already existing channels with biomolecules enabling cell guidance (not shown). Planar processing utilizes a PDMS stamp to generate layers of channels in hydrogels. This multi-step-procedure is preferably used for establishing perfusion networks in hydrogels. Hollow channels increase control of endothelial cell distribution and improve mass transport. Endothelialized channels promote vessel ingrowth and anastomosis with host vasculature leading to better integration into host tissue upon implantation.

The use of removable spacers can guarantee a defined channel structure and geometry such as channel diameter to yield adequate conditions of mass transport throughout a hydrogel (Moore et al., 2006; Bagnaninchi et al., 2007; Huang et al., 2013). This technique is also frequently used for neural tissue engineering to assist nerve guidance (Nectow et al., 2012) and a few studies published recently show their potential applicability for endothelialization of hydrogel channels in silk scaffolds (Wray et al., 2012; Rnjak-Kovacina et al., 2013). However, mechanically removable spacers are primarily suited for creating unbranched structures, which do not resemble the *in vivo* situation (Figure 1). Nevertheless, it has been demonstrated that effective endothelialization of channels can be achieved by injecting a cell-laden hydrogel into hollow channels of a solid scaffold. Endothelial cells align at the inner surface of channels while supporting cells present in the surrounding accumulate around them (Wray et al., 2012). However, the mere presence of supporting cells such as fibroblasts in the bulk can suffice to improve vascularization and integration of implanted scaffolds *in vivo* presumably as these channels can enhance nutrient delivery (Rnjak-Kovacina et al., 2013). Indeed, it has been shown that enhanced vascularization of an *in vitro*-generated construct is achieved by sprouting of embedded endothelial cells toward pre-existing channels perfused with nutrients (Sakaguchi et al., 2013). Spacers can furthermore be pre-seeded with endothelial cells, which are then released to attach on the inner surface of the hydrogel channel using alternating electrical potentials and zwitterionic ions attached the cell surface (Sadr et al., 2011).

Instead of mechanical removal, spacers can also be crafted out of sacrificial materials to be used to create template-based structures within hydrogels. The basic principle is the incorporation of certain sacrificial structures into a hydrogel and their removal by exploiting its specific physical properties such as melting point or solubility (Huang et al., 2011). The feasibility and benefit of this technique for vascularization has been demonstrated in various hydrogels, e.g., with carbohydrate glass as sacrificial element (Miller et al., 2012). By embedding gelatine structures in collagen or fibrin gels, Golden and Tien were able to generate microchannel structures after a melting and flushing step to remove gelatine (Golden and Tien, 2007). When seeding endothelial cells into these channels, they furthermore observed alignment of cells when subjected to shear stress. More recently, multi-layered channels have been fabricated in collagen gels using the same technique (Lee et al., 2010). These endothelialized structures have been shown to improve survival of cells embedded in the matrix. Additionally, endothelial cells can sprout from this channel into the surrounding space and form a lumen (Lee et al., 2014). However, gelatine can also serve as hydrogel scaffold having sacrificial alginate fibers embedded. Alginate polymerizes reversibly after addition of calcium and it can thus be easily removed using calcium binding agents such as ethylene diamine tetraacetic acid (EDTA) (Hammer et al., 2013). Recently, endothelialized channels have been engineered by dissolving alginate fibers embedded in cell-laden fibrin gels. Perfusion of these hollow channels resulted in elongation of endothelial cells in the lumen (Vollert et al., 2014). Phosphate-based glass fibers have been shown to be a suitable degradable material for generating microchannels in collagen scaffolds as altering their chemistry can initiate degradation within minutes (Nazhat et al., 2007).

## 3D Printing

Additive manufacturing technologies, often referred to as 3D printing, are computer-assisted methods attractive for fabrication of porous architectures of almost any complexity. Some of these methods, including sterelithography and digital light processing, employ light-induced polymerization in order to define precise patterns in monomer solutions (Skoog et al., 2013). 3D structures are produced in a layer-by-layer fashion. The important advantage is the capability to produce hollow channels interconnecting between layers and complex networks thus enabling branching out of the plane (Figure 1). Recently different 3D printing methods were used to create microstructures in hydrogels. A few noteworthy studies relevant for vascularization strategies are presented in this review. For more information, the reader is directed to recently published work focusing on these techniques and their advantages in tissue engineering (Ovsianikov et al., 2012; Skoog et al., 2013; Murphy and Atala, 2014; Narayan, 2014). Photopolymerization can accurately and quickly generate patterns within a hydrogel, and even to trap cells in a 3D matrix and thus enable engineering of 3D tissue models (Ovsianikov et al., 2014). The proof of concept and biocompatibility of this method has been demonstrated in an attempt to engineer heart valves using interstitial cells encapsulated in gelatine (Benton et al., 2009). Furthermore, laser-based lithography can functionalize hydrogels to direct cell growth, a technique known as photocoupling (DeForest and Anseth, 2011). This method would allow precise tissue design as human fibroblasts have been shown to migrate only toward arginine-glycine-aspartate (RGD)-functionalized areas inside the scaffold while leaving others empty (Lee et al., 2008). This has been functionally assessed by determining the growth of rat dorsal root ganglia toward a patterned channel (Luo and Shoichet, 2004). Additionally, it has been shown that multiple growth factors can be patterned into a hydrogel structure (Wylie et al., 2011). These techniques allow to accurately control directional growth of cells within a scaffold. Moreover, to entirely endothelialize a scaffold it has been shown that interconnectivity of hydrogel pores is beneficial for the proliferation of uniformly seeded cells thus paving a way toward vascularized artificial constructs (Gauvin et al., 2012). However, despite all advantages and efforts to optimize materials, there is still the need for adequate reagents for cell encapsulation. Many photoinitiators used for free-radical-polymerization show a considerable photo-toxicity, which needs to be reduced to accurately generate cell-compatible photo-hydrogels (Ovsianikov et al., 2014). Although most techniques require cells to be incorporated after fabrication, the feasibility of 3D printing to deposit cells and scaffold material simultaneously into a microstructure has also been demonstrated (Gruene et al., 2011).

## Planar Processing

Another simple way of creating a layer of microchannels is by using soft lithography micromolding. The most important feature is the planarity of this method, which makes it primarily suited for designing microfluidic channels in hydrogels (Annabi et al., 2010; Bhatia and Ingber, 2014). Most applications involve a photo-resistant silicon wafer used for UV-light-initiated polymerization together with a photomask having a selected pattern incorporated (Figure 1). Polydimethylsiloxane (PDMS) is then poured onto a wafer following curing and removal of the PDMS mold or stamp. The desired polymer is then poured onto the PDMS stamp, polymerization is initiated and the polymer is separated from the stamp. Channels are finally enclosed by curing a solid hydrogel layer on top of the produced scaffold. Perfusion of the created channels is required to ensure viability of cells embedded in the hydrogel (Ling et al., 2007). More importantly, microvasculature can only develop in the proximity of channels supplying endothelial cells with nutrients as shown in experiments with molded PEG hydrogels (Cuchiara et al., 2012). It has been reported that long-term culture and growth of endothelial cells is possible when cells are seeded into perfused hydrogel channels, a key step toward development of *in vitro* engineered vasculature (Shin et al., 2004). Additionally, endothelial cells seeded into hydrogel channels are self-aligning under static conditions demonstrating the influence certain microstructures can have on cell morphogenesis (Aubin et al., 2010). In a recent study, engineered microvascular networks have been established in collagen scaffolds using soft lithography (Zheng et al., 2012). A similar result has also been reported in molded channels filled with endothelial cell-laden collagen gels where formation of capillaries was observed within 48 h of incubation (Raghavan et al., 2010). Moreover, combinatorial approaches using micromolding together with another processing technique can be used to engineer structured hydrogels. Using sacrificial elements in combination with micromolding have been shown to accurately and efficiently generate 3D networks of perfusable channels (Golden and Tien, 2007). A multi-channeled device having endothelial cells separated from co-cultured fibroblasts has been developed to study angiogenesis and vasculogenesis on a microscale. The resulting vascular networks are perfusable and suitable to study endothelial sprouting and cancer metastasis (Kim et al., 2013). Recently, another method using bioprinted channel networks, subsequent embedding in various hydrogel materials and injection of human umbilical vein endothelial cells (HUVEC) was reported to result in a cell monolayer inside a perfused microvessel (Bertassoni et al., 2014). While most groups create structures within hydrogels, it has also been reported that microstructures can be coated with a modified gelatine resulting in a hydrogel channel (Annabi et al., 2013). Additionally, hydrogels containing microstructures and embedded cells can also be sequentially assembled to generate a branched channel network (Du et al., 2011). Interestingly, a recent study suggests to incorporate empty draining channels similar to lymphatic vessels in addition to vascularized structures as it increases vascular adhesion and stabilizes perfusion rate in dense hydrogels (Wong et al., 2013).

## Miscellaneous

An interesting approach has been reported by the group of Dror Seliktar. By using PEGylated fibrinogen, it has been demonstrated that patterns can be accurately and quickly produced through photoablation (Sarig-Nadir et al., 2009). Although this method achieves similar result as 3D printing, creation of hollow channels does not necessarily rely on a specific photochemistry or material design. These created channels have been shown to facilitate directed growth of neural cells. However, a potential applicability for channel endothelialization is given. Recently, a report demonstrated an interesting approach using bioprinting for cell and material deposition to establish structured hydrogels (Kolesky et al., 2014). Microvasculature composed of HUVECs together with channels containing different fibroblast types were bioprinted in a gelatine hydrogel. These engineered capillaries were perfused with media ensuring survival for at least 7 days of all cells incorporated. As most vascularized tissues are heterogenous, selective deposition of cells and materials is an attractive tool to generate vascularized tissue-engineered constructs. An interesting technology to manipulate whole cell sheets has also been reported, which could be useful to seed whole layers of endothelial cells into a prepared channel (Asakawa et al., 2010). Additionally, multiple sheets comprised out of endothelial cells and mural cells can be manipulated and seeded onto certain surfaces. This can potentially be used to prepare adequate cell sheet linings to engineer blood vessel walls *in vitro*.

## Comparison of Different Methods

A comparison of techniques presented is summarized in Table 1. The removal of intermediate spacers is an easy-to-use tool to generate interconnected structures in hydrogels. Channels of various geometries can be created using the described methods to increase mass transport and promote cell distribution. In contrast to 3D printing and most molding applications, these technologies can work with natural unmanipulated polymers as already existing physical properties such as mechanical stiffness or melting point are exploited. Still, non-sacrificial spacers do not allow branching in contrast to sacrificial elements. Nevertheless, it is important to state that both spacer approaches utilize equipment virtually any research lab has easily access to. The spatial resolution for the methods presented has been reported to range from 20 to 50 μm for both intermediate spacers (Golden and Tien, 2007; Sakaguchi et al., 2013) and micromolded channels (Raghavan et al., 2010). 3D printing technologies, in particular multiphoton processing, have been demonstrated to yield spatial resolutions in the micrometer range, which are superior to other approaches (Ovsianikov et al., 2014). The accuracy and speed of this technique has been demonstrated in an experiment where a whole organism (*Caenorhabditis elegans*) has been encaged by rapid processing of the surrounding hydrogel (Torgersen et al., 2012). Yet, most applications rely on photochemistry or certain modified materials. A common issue with free-radical based photopolymerization is possible photoxicity, which can result in cell damage (Ovsianikov et al., 2014). Similar to incorporation of constructs made out of sacrificial elements, 3D printing can facilitate branching out of the plane, a feature that could not be shown for micromolding. However, using the same micromolded replica in experiments generates a remarkable reproducibility, which cannot be compared to reproducibility of other approaches. Nevertheless, as shown in Table S1 in Supplementary Material the use of removable spacers can successfully create channel structures in a variety of hydrogel materials. Moreover, reference values for material stiffness demonstrate that channels can even be engineered in materials having a low stiffness such as collagen or fibrin at low concentrations. Still, the method of choice relies on the scientific question and parameters that need to be tested and, for some approaches, on the equipment available.

**Table 1:** **Methods used to create channels in hydrogels and their advantages and disadvantages**.

Method	Pro	Contra
Non-sacrificial spacer	Defined channel geometry, easy-to-use	Almost no branching possible, removal of spacers can destroy ultrastructure, resulting channels have a rather large size
Sacrificial spacer	Good technique to create interconnected channels, virtually any template structure is suitable	Materials used need to have distinct physical properties in order to be removed, e.g., melting point, and at the same time the removal should not influence the hydrogel itself
Photopolymerization-based 3D printing	A group of fast, accurate methods to generate any designed microstructure in a hydrogel, micrometer resolution	Free-radical-polymerization can potentially damage cells, specialized equipment, and materials are necessary
Planar	Template can be used repeatedly, suitable to generate highly organized perfusion channel network of defined geometry	Multi-step stacking procedure can be time-consuming

## The Importance of the Endothelial Cell Type Used

For every approach that aims at promoting vascularization, it is inevitable to consider endothelial cell biology. While a detailed discussion of the adequate cell source would go beyond the scope of this review, a few key points are highlighted in this section. The endothelium is a heterogenous organ where cells have major phenotypic differences dependent on the organ, location, vessel, and vessel size (Aird, 2012). Umbilical veins are a readily available “waste material” and used for decades to isolate endothelial cells thereof. Currently, these cells represent the golden standard in experiments using endothelial cells (Baiguera and Ribatti, 2013). However, autologous use of HUVECs for tissue engineering is only possible with limited success. Adult microvascular endothelial cells would be an ideal cell source for vascularization strategies of hydrogels. Unfortunately, their proliferative potential is often impaired and harvesting of these cells results in donor site morbidity (Poh et al., 2005). Endothelial progenitor cells (EPC) isolated from donated blood serve as an ideal cell source for clinical applications in regenerative medicine as they can be harvested, isolated and re-implanted in an autologous manner (Fuchs et al., 2010; Badylak et al., 2012). However, multiple clones for EPC mass production have to be obtained through isolation in order to reach clinically relevant cell numbers (Reinisch et al., 2009). Still, these cells can be co-cultured with other autologously isolated cells such as adult stem cells promoting vascularization of hydrogels (Holnthoner et al., 2012; Rohringer et al., 2014). Additionally, many protocols for isolation and expansion of these cells have been developed demonstrating that these cells can even be used for proof-of-principle tests.

## Concluding Remarks

In most soft tissue engineering applications, implantation of inadequately vascularized hydrogels will result in hypoxia or even anoxia and cell death. To address this problem, fast ingrowth of host blood vessels into a scaffold needs to be considered in the design of tissue-engineered constructs. Endothelialized channels and interconnected structures aim to resemble native microvasculature thus promoting survival and function of cells embedded in an implant through formation of anastomoses with host tissue. A variety of reproducible and accurate techniques exists to generate channeled hydrogels. Despite all efforts to develop feasible methods to alter microstructures, only a few studies incorporate endothelial cells in their hydrogels. Unfortunately, the majority of publications show a proof-of-principle rather than demonstrating its applicability for vascularization using an adequate cell source. Microvascular endothelial cells would be ideal to be embedded in engineered microvessels while macrovascular endothelial cells such as HUVEC have their limitations but are suitable for preliminary tests. Additionally, EPCs from peripheral or umbilical vein blood offer a clinically relevant cell source as they can be harvested minimally invasively and used autologously. Despite all these intriguing advantages, this cell type is currently strongly neglected in biofabrication. Engineering methods are improving rapidly and more emphasis needs to be put on the biology of microvasculature when designing *in vitro* constructs. Therefore, successful hydrogel integration and cell survival can be achieved using accurate and feasible engineering techniques with equal consideration of vascular biology.

## Conflict of Interest Statement

The authors declare that the research was conducted in the absence of any commercial or financial relationships that could be construed as a potential conflict of interest.

## Supplementary Material

The Supplementary Material for this article can be found online at http://www.frontiersin.org/Journal/10.3389/fbioe.2014.00052/abstract
