# Supplementary material for: Connections Matter: Channeled Hydrogels to Improve Vascularization
Source: Front Bioeng Biotechnol. 2014 Nov 14;2:52. doi: 10.3389/fbioe.2014.00052 (PMC4231943; doi:10.3389/fbioe.2014.00052)
Supplement: Supplementary file 1 [file Table_1.PDF]

Supplementary Material: Connections matter: channeled hydrogels to improve vascularization

Severin Muehleder, Aleksandr Ovsianikov, Johannes Zipperle, Heinz Redl, Wolfgang Holnthoner

1. Supplementary Table

Table S1: List of hydrogel materials including working concentrations in which channels were incorporated in by using removable spacers, laser-assisted 3D printing or planar processing to improve vascularization.

	Method			
**Hydrogels based on**	Removable spacer	Laser-assisted 3D printing	Planar processing	**Stiffness G' [kPa]**
gelatin	**10 %**[1,2]	**3.25-10 %**[12,13,14]	**5-12. 5%**[20,21,22,23]	16-19 (15 %)[31]
PEG	**5, 10 or 20 % (6 and 35 kDa)**[3]	**10 % (10,000 g/mol)**[15,16]	**5-20 %**[21,24,25]	80-100 (6000 g/mol)[32]
collagen	**2.3 -21 mg/mL**[4,5,6,7,8]		**6-10 mg/mL**[26]	0.180-0.250 (3 mg/mL)[33]
agarose	**2 %**[3]	**0.5 – 1 %**[17,18]	**3 %**[27]	0.120-0.140 (2 %)[34]
fibrin	**5-50 mg/mL fibrinogen; 3-3.4 U/mL thrombin**[3,5,9]		**2.5-50 mg/mL fibrinogen; 0.5-3.4 U/mL thrombin**[5,28,29]	0.120-0.140 (6 mg/mL)[35]
Matrigel	**8-11 mg/mL**[3]			0.02 (4 mg/mL)[36]
silk	**2-8 %**[10,11]			18-20 (25-30 %[37]
alginate	**2 %**[3]			3 (3 %)[38]
hyaluronic acid (HA)/fibrin		**1% HA/ 13.3 mg/mL fibrinogen/ 24 U/mL thrombin**[19]		N/A
hyaluronic acid/gelatin			**1-3 % (HA)/2.5-7.5 % (gelatin)**[30]	N/A

[1](Sadr et al., 2011), [2](Hammer et al., 2013), [3](Miller et al., 2012), [4](Sakaguchi et al., 2013), [5](Golden and Tien, 2007), [6](Lee et al., 2010), [7](Lee et al., 2014), [8](Nazhat et al., 2007), [9](Vollert et al., 2014), [10](Rnjak-Kovacina et al., 2013), [11](Wray et al., 2012), [12](Benton et al., 2009), [13](Ovsianikov et al., 2014), [14](Gauvin et al., 2012), [15](DeForest and Anseth, 2011), [16](Lee et al., 2008), [17](Luo and Shoichet, 2004), [18](Wylie et al., 2011), [19](Gruene et al., 2011), [20](Aubin et al., 2010), [21](Bertassoni et al., 2014), [22](Annabi et al., 2013), [23](Wang et al., 2014), [24](Cuchiara et al., 2012), [25](Du et al., 2011), [26](Zheng et al., 2012), [27](Ling et al., 2007), [28](Kim et al., 2013), [29](Wong et al., 2013), [30](Kageyama et al., 2014), [31](Xing et al., 2014), [32](Schmocker et al., 2014), [33](Arevalo et al., 2010), [34](Balgude et al., 2001), [35](Ryan et al., 1999), [36](Norman et al., 2007), [37](Holland et al., 2006), [38](West et al., 2007)

2. References

Aird, W. C. (2012). Endothelial cell heterogeneity. *Cold Spring Harb. Perspect. Med.* 2, a006429. doi:10.1101/cshperspect.a006429.

Annabi, N., Nichol, J. W., Zhong, X., Ji, C., Koshy, S., Khademhosseini, A., and Dehghani, F. (2010). Controlling the porosity and microarchitecture of hydrogels for tissue engineering. *Tissue Eng. Part B. Rev.* 16, 371–83. doi:10.1089/ten.TEB.2009.0639.

Annabi, N., Selimović, Š., Acevedo Cox, J. P., Ribas, J., Afshar Bakooshli, M., Heintze, D., Weiss, A. S., Cropek, D., and Khademhosseini, A. (2013). Hydrogel-coated microfluidic channels for cardiomyocyte culture. *Lab Chip* 13, 3569–77. doi:10.1039/c3lc50252j.

Arevalo, R. C., Urbach, J. S., and Blair, D. L. (2010). Size-dependent rheology of type-I collagen networks. *Biophys. J.* 99, L65–7. doi:10.1016/j.bpj.2010.08.008.

Asakawa, N., Shimizu, T., Tsuda, Y., Sekiya, S., Sasagawa, T., Yamato, M., Fukai, F., and Okano, T. (2010). Pre-vascularization of in vitro three-dimensional tissues created by cell sheet engineering. *Biomaterials* 31, 3903–9. doi:10.1016/j.biomaterials.2010.01.105.

Aubin, H., Nichol, J. W., Hutson, C. B., Bae, H., Sieminski, A. L., Cropek, D. M., Akhyari, P., and Khademhosseini, A. (2010). Directed 3D cell alignment and elongation in microengineered hydrogels. *Biomaterials* 31, 6941–6951. doi:10.1016/j.biomaterials.2010.05.056.

Badylak, S. F., Weiss, D. J., Caplan, A., and Macchiarini, P. (2012). Engineered whole organs and complex tissues. *Lancet* 379, 943–52. doi:10.1016/S0140-6736(12)60073-7.

Bagnaninchi, P. O., Yang, Y., Zghoul, N., Maffulli, N., Wang, R. K., and Haj, a J. El (2007). Chitosan microchannel scaffolds for tendon tissue engineering characterized using optical coherence tomography. *Tissue Eng.* 13, 323–31. doi:10.1089/ten.2006.0168.

Baiguera, S., and Ribatti, D. (2013). Endothelialization approaches for viable engineered tissues. *Angiogenesis* 16, 1–14. doi:10.1007/s10456-012-9307-8.

Balgude, A. P., Yu, X., Szymanski, A., and Bellamkonda, R. V (2001). Agarose gel stiffness determines rate of DRG neurite extension in 3D cultures. *Biomaterials* 22, 1077–84.

Benton, J. A., DeForest, C. A., Vivekanandan, V., and Anseth, K. S. (2009). Photocrosslinking of gelatin macromers to synthesize porous hydrogels that promote valvular interstitial cell function. *Tissue Eng. Part A* 15, 3221–30. doi:10.1089/ten.TEA.2008.0545.

Bertassoni, L. E., Cecconi, M., Manoharan, V., Nikkhah, M., Hjortnaes, J., Cristino, A. L., Barabaschi, G., Demarchi, D., Dokmeci, M. R., Yang, Y., et al. (2014). Hydrogel bioprinted microchannel networks for vascularization of tissue engineering constructs. *Lab Chip*, 2202–2211. doi:10.1039/c4lc00030g.

Bhatia, S. N., and Ingber, D. E. (2014). Microfluidic organs-on-chips. *Nat. Biotechnol.* 32, 760–772. doi:10.1038/nbt.2989.

Cuchiara, M. P., Gould, D. J., McHale, M. K., Dickinson, M. E., and West, J. L. (2012). Integration of Self-Assembled Microvascular Networks with Microfabricated PEG-Based Hydrogels. *Adv. Funct. Mater.* 22, 4511–4518. doi:10.1002/adfm.201200976.

DeForest, C., and Anseth, K. (2011). Cytocompatible click-based hydrogels with dynamically tunable properties through orthogonal photoconjugation and photocleavage reactions. *Nat. Chem.* 3, 925–931. doi:10.1038/NCHEM.1174.

Du, Y., Ghodousi, M., Qi, H., Haas, N., Xiao, W., and Khademhosseini, A. (2011). Sequential assembly of cell-laden hydrogel constructs to engineer vascular-like microchannels. *Biotechnol. Bioeng.* 108, 1693–703. doi:10.1002/bit.23102.

Fortelny, R. H., Petter-Puchner, A. H., Glaser, K. S., and Redl, H. (2012). Use of fibrin sealant (Tisseel/Tissucol) in hernia repair: a systematic review. *Surg. Endosc.* 26, 1803–12. doi:10.1007/s00464-012-2156-0.

Fuchs, S., Dohle, E., Kolbe, M., and Kirkpatrick, C. J. (2010). Outgrowth endothelial cells: sources, characteristics and potential applications in tissue engineering and regenerative medicine. *Adv. Biochem. Eng. Biotechnol.* 123, 201–17. doi:10.1007/10_2009_65.

Gauvin, R., Chen, Y., Lee, J., and Soman, P. (2012). Microfabrication of complex porous tissue engineering scaffolds using 3D projection stereolithography. *Biomaterials* 33, 3824–3834. doi:10.1016/j.biomaterials.2012.01.048.Gauvin.

Geckil, H., Xu, F., Zhang, X., Moon, S., and Demirci, U. (2010). Engineering hydrogels as extracellular matrix mimics. *Nanomedicine (Lond).* 5, 469–84. doi:10.2217/nnm.10.12.

Golden, A., and Tien, J. (2007). Fabrication of microfluidic hydrogels using molded gelatin as a sacrificial element. *Lab Chip*, 720–725. doi:10.1039/b618409j.

Gruene, M., Pflaum, M., Hess, C., Diamantouros, S., Schlie, S., Deiwick, A., Koch, L., Wilhelmi, M., Jockenhoevel, S., Haverich, A., et al. (2011). Laser printing of three-dimensional multicellular arrays for studies of cell-cell and cell-environment interactions. *Tissue Eng. Part C. Methods* 17, 973–82. doi:10.1089/ten.TEC.2011.0185.

Hammer, J., Han, L., Tong, X., and Yang, F. (2013). A Facile Method to Fabricate Hydrogels with Microchannel-Like Porosity for Tissue Engineering. 20, 24–27. doi:10.1089/ten.tec.2013.0176.

Holland, C., Terry, a E., Porter, D., and Vollrath, F. (2006). Comparing the rheology of native spider and silkworm spinning dope. *Nat. Mater.* 5, 870–4. doi:10.1038/nmat1762.

Holnthoner, W., Hohenegger, K., Husa, A.-M., Muehleder, S., Meinl, A., Peterbauer-Scherb, A., and Redl, H. (2012). Adipose-derived stem cells induce vascular tube formation of outgrowth endothelial cells in a fibrin matrix. *J. Tissue Eng. Regen. Med.* doi:10.1002/term.1620.

Huang, G., Wang, S., He, X., Zhang, X., Lu, T. J., and Xu, F. (2013). Helical spring template fabrication of cell-laden microfluidic hydrogels for tissue engineering. *Biotechnol. Bioeng.* 110, 980–9. doi:10.1002/bit.24764.

Huang, G. Y., Zhou, L. H., Zhang, Q. C., Chen, Y. M., Sun, W., Xu, F., and Lu, T. J. (2011). Microfluidic hydrogels for tissue engineering. *Biofabrication* 3, 012001. doi:10.1088/1758-5082/3/1/012001.

Kageyama, T., Kakegawa, T., Osaki, T., Enomoto, J., Ito, T., Nittami, T., and Fukuda, J. (2014). Rapid engineering of endothelial cell-lined vascular-like structures in in situ crosslinkable hydrogels. *Biofabrication* 6, 25006.

Kim, S., Lee, H., Chung, M., and Jeon, N. L. (2013). Engineering of functional, perfusable 3D microvascular networks on a chip. *Lab Chip* 13, 1489–500. doi:10.1039/c3lc41320a.

Kirkpatrick, C. J., Fuchs, S., and Unger, R. E. (2011). Co-culture systems for vascularization — Learning from nature. *Adv. Drug Deliv. Rev.* 63, 291–299. doi:http://dx.doi.org/10.1016/j.addr.2011.01.009.

Kirschner, C. M., and Anseth, K. S. (2013). Hydrogels in Healthcare: From Static to Dynamic Material Microenvironments. *Acta Mater.* 61, 931–944. doi:10.1016/j.actamat.2012.10.037.

Ko, H. C. H., Milthorpe, B. K., and McFarland, C. D. (2007). Engineering thick tissues--the vascularisation problem. *Eur. Cell. Mater.* 14, 1–18; discussion 18–9.

Kolesky, D. B., Truby, R. L., Gladman, a S., Busbee, T. a, Homan, K. a, and Lewis, J. a (2014). 3D Bioprinting of Vascularized, Heterogeneous Cell-Laden Tissue Constructs. *Adv. Mater.* 26, 3124–30. doi:10.1002/adma.201305506.

Lee, S.-H., Moon, J. J., and West, J. L. (2008). Three-dimensional micropatterning of bioactive hydrogels via two-photon laser scanning photolithography for guided 3D cell migration. *Biomaterials* 29, 2962–8. doi:10.1016/j.biomaterials.2008.04.004.

Lee, V. K., Kim, D. Y., Ngo, H., Lee, Y., Seo, L., Yoo, S.-S., Vincent, P. a, and Dai, G. (2014). Creating perfused functional vascular channels using 3D bio-printing technology. *Biomaterials* 35, 8092–8102. doi:10.1016/j.biomaterials.2014.05.083.

Lee, W., Lee, V., Polio, S., Keegan, P., Lee, J.-H., Fischer, K., Park, J.-K., and Yoo, S.-S. (2010). On-demand three-dimensional freeform fabrication of multi-layered hydrogel scaffold with fluidic channels. *Biotechnol. Bioeng.* 105, 1178–86. doi:10.1002/bit.22613.

Levenberg, S., Rouwkema, J., Macdonald, M., Garfein, E. S., Kohane, D. S., Darland, D. C., Marini, R., van Blitterswijk, C. A., Mulligan, R. C., D’Amore, P. A., et al. (2005). Engineering vascularized skeletal muscle tissue. *Nat Biotech* 23, 879–884.

Ling, Y., Rubin, J., Deng, Y., Huang, C., Demirci, U., Karp, J. M., and Khademhosseini, A. (2007). A cell-laden microfluidic hydrogel. *Lab Chip* 7, 756–62. doi:10.1039/b615486g.

Luo, Y., and Shoichet, M. S. (2004). A photolabile hydrogel for guided three-dimensional cell growth and migration. *Nat. Mater.* 3, 249–53. doi:10.1038/nmat1092.

Marra, K. G., Ph, D., and Kaplan, D. L. (2012). Biomaterials for the Development of Peripheral Nerve. 18, 40–50. doi:10.1089/ten.teb.2011.0240.

Miller, J. S., Stevens, K. R., Yang, M. T., Baker, B. M., Nguyen, D.-H. T., Cohen, D. M., Toro, E., Chen, A. a, Galie, P. a, Yu, X., et al. (2012). Rapid casting of patterned vascular networks for perfusable engineered three-dimensional tissues. *Nat. Mater.* 11, 768–74. doi:10.1038/nmat3357.

Moore, M. J., Friedman, J. a, Lewellyn, E. B., Mantila, S. M., Krych, A. J., Ameenuddin, S., Knight, A. M., Lu, L., Currier, B. L., Spinner, R. J., et al. (2006). Multiple-channel scaffolds to promote spinal cord axon regeneration. *Biomaterials* 27, 419–29. doi:10.1016/j.biomaterials.2005.07.045.

Murphy, S. V, and Atala, A. (2014). 3D bioprinting of tissues and organs. *Nat. Biotechnol.* 32, 773–785. doi:10.1038/nbt.2958.

Narayan, R. J. (2014). *Rapid Prototyping of Biomaterials*. Elsevier doi:10.1016/B978-0-85709-599-2.50016-7.

Nazhat, S. N., Neel, E. A. A., Kidane, A., Ahmed, I., Hope, C., Kershaw, M., Lee, P. D., Stride, E., Saffari, N., Knowles, J. C., et al. (2007). Controlled microchannelling in dense collagen scaffolds by soluble phosphate glass fibers. *Biomacromolecules* 8, 543–51. doi:10.1021/bm060715f.

Nicolson, P. C., and Vogt, J. (2001). Soft contact lens polymers: an evolution. *Biomaterials* 22, 3273–83.

Norman, J. J., Collins, J. M., Sharma, S., Russell, B., and Desai, T. a. (2007). Microstructures in 3D Biological Gels Affect Cell Proliferation. *Tissue Eng.* 00, 110306233438005. doi:10.1089/ten.2007.0077.

Novosel, E., Kleinhans, C., and Kluger, P. (2011). Vascularization is the key challenge in tissue engineering. *Adv. Drug Deliv. Rev.* 63, 300–311. doi:10.1016/j.addr.2011.03.004.

Ovsianikov, A., Mironov, V., Stampfl, J., and Liska, R. (2012). Engineering 3D cell-culture matrices: multiphoton processing technologies for biological and tissue engineering applications. *Expert Rev. Med. Devices* 9, 613–633. doi:10.1586/erd.12.48.

Ovsianikov, A., Mühleder, S., Torgersen, J., Li, Z., Qin, X.-H., Van Vlierberghe, S., Dubruel, P., Holnthoner, W., Redl, H., Liska, R., et al. (2014). Laser photofabrication of cell-containing hydrogel constructs. *Langmuir* 30, 3787–94. doi:10.1021/la402346z.

Poh, M., Boyer, M., Solan, A., and Dahl, S. (2005). Blood vessels engineered from human cells. *Lancet* 365, 2122–2124.

Raghavan, S., Nelson, C. M., Baranski, J. D., Lim, E., and Chen, C. S. (2010). Geometrically controlled endothelial tubulogenesis in micropatterned gels. *Tissue Eng. Part A* 16, 2255–63. doi:10.1089/ten.TEA.2009.0584.

Reinisch, A., Hofmann, N. a, Obenauf, A. C., Kashofer, K., Rohde, E., Schallmoser, K., Flicker, K., Lanzer, G., Linkesch, W., Speicher, M. R., et al. (2009). Humanized large-scale expanded endothelial colony-forming cells function in vitro and in vivo. *Blood* 113, 6716–25. doi:10.1182/blood-2008-09-181362.

Rnjak-Kovacina, J., Wray, L. S., Golinski, J. M., and Kaplan, D. L. (2013). Arrayed Hollow Channels in Silk-Based Scaffolds Provide Functional Outcomes for Engineering Critically Sized Tissue Constructs. *Adv. Funct. Mater.*, n/a–n/a. doi:10.1002/adfm.201302901.

Rohringer, S., Hofbauer, P., Schneider, K. H., Husa, A.-M., Feichtinger, G., Peterbauer-Scherb, A., Redl, H., and Holnthoner, W. (2014). Mechanisms of vasculogenesis in 3D fibrin matrices mediated by the interaction of adipose-derived stem cells and endothelial cells. *Angiogenesis*. doi:10.1007/s10456-014-9439-0.

Rouwkema, J., Rivron, N. C., and van Blitterswijk, C. A. (2008). Vascularization in tissue engineering. *Trends Biotechnol.* 26, 434–41. doi:10.1016/j.tibtech.2008.04.009.

Roy, T. D., Simon, J. L., Ricci, J. L., Rekow, E. D., Thompson, V. P., and Parsons, J. R. (2003). Performance of degradable composite bone repair products made via three-dimensional fabrication techniques. *J. Biomed. Mater. Res. A* 66, 283–91. doi:10.1002/jbm.a.10582.

Ryan, E. A., Mockros, L. F., Weisel, J. W., and Lorand, L. (1999). Structural origins of fibrin clot rheology. *Biophys. J.* 77, 2813–26.

Sadr, N., Zhu, M., Osaki, T., Kakegawa, T., Yang, Y., Moretti, M., Fukuda, J., and Khademhosseini, A. (2011). SAM-based cell transfer to photopatterned hydrogels for microengineering vascular-like structures. *Biomaterials* 32, 7479–90. doi:10.1016/j.biomaterials.2011.06.034.

Sakaguchi, K., Shimizu, T., Horaguchi, S., Sekine, H., Yamato, M., Umezu, M., and Okano, T. (2013). In vitro engineering of vascularized tissue surrogates. *Sci. Rep.* 3, 1316. doi:10.1038/srep01316.

Sarig-Nadir, O., Livnat, N., Zajdman, R., Shoham, S., and Seliktar, D. (2009). Laser photoablation of guidance microchannels into hydrogels directs cell growth in three dimensions. *Biophys. J.* 96, 4743–52. doi:10.1016/j.bpj.2009.03.019.

Schmocker, A., Khoushabi, A., Schizas, C., Bourban, P.-E., Pioletti, D. P., and Moser, C. (2014). Photopolymerizable hydrogels for implants: Monte-Carlo modeling and experimental in vitro validation. *J. Biomed. Opt.* 19, 35004. doi:10.1117/1.JBO.19.3.035004.

Seliktar, D. (2012). Designing cell-compatible hydrogels for biomedical applications. *Science* 336, 1124–8. doi:10.1126/science.1214804.

Shin, M., Matsuda, K., Ishii, O., Terai, H., Kaazempur-Mofrad, M., Borenstein, J., Detmar, M., and

Vacanti, J. P. (2004). Endothelialized networks with a vascular geometry in microfabricated poly(dimethyl siloxane). *Biomed. Microdevices* 6, 269–78. doi:10.1023/B:BMMD.0000048559.29932.27.

Skoog, S. a, Goering, P. L., and Narayan, R. J. (2013). Stereolithography in tissue engineering. *J. Mater. Sci. Mater. Med.*, 845–856. doi:10.1007/s10856-013-5107-y.

Torgersen, J., Ovsianikov, A., Mironov, V., Pucher, N., Qin, X., Li, Z., Cicha, K., Machacek, T., Liska, R., Jantsch, V., et al. (2012). Photo-sensitive hydrogels for three-dimensional laser microfabrication in the presence of whole organisms. *J. Biomed. Opt.* 17, 105008. doi:10.1117/1.JBO.17.10.105008.

Vollert, I., Seiffert, M., Bachmair, J., Sander, M., Eder, A., Conradi, L., Vogelsang, A., Schulze, T., Uebeler, J., Holnthoner, W., et al. (2014). In vitro perfusion of engineered heart tissue through endothelialized channels. *Tissue Eng. Part A* 20, 854–63. doi:10.1089/ten.TEA.2013.0214.

Wang, X.-Y., Jin, Z.-H., Gan, B.-W., Lv, S.-W., Xie, M., and Huang, W.-H. (2014). Engineering interconnected 3D vascular networks in hydrogels using molded sodium alginate lattice as the sacrificial template. *Lab Chip*. doi:10.1039/c4lc00069b.

West, E. R., Xu, M., Woodruff, T. K., and Shea, L. D. (2007). Physical properties of alginate hydrogels and their effects on in vitro follicle development. *Biomaterials* 28, 4439–48. doi:10.1016/j.biomaterials.2007.07.001.

Wong, K. H. K., Truslow, J. G., Khankhel, A. H., Chan, K. L. S., and Tien, J. (2013). Artificial lymphatic drainage systems for vascularized microfluidic scaffolds. *J. Biomed. Mater. Res. A* 101, 2181–90. doi:10.1002/jbm.a.34524.

Wray, L. S., Rnjak-Kovacina, J., Mandal, B. B., Schmidt, D. F., Gil, E. S., and Kaplan, D. L. (2012). A silk-based scaffold platform with tunable architecture for engineering critically-sized tissue constructs. *Biomaterials* 33, 9214–24. doi:10.1016/j.biomaterials.2012.09.017.

Wylie, R. G., Ahsan, S., Aizawa, Y., Maxwell, K. L., Morshead, C. M., and Shoichet, M. S. (2011). Spatially controlled simultaneous patterning of multiple growth factors in three-dimensional hydrogels. *Nat. Mater.* 10, 799–806. doi:10.1038/nmat3101.

Xing, Q., Yates, K., Vogt, C., Qian, Z., Frost, M. C., and Zhao, F. (2014). Increasing mechanical strength of gelatin hydrogels by divalent metal ion removal. *Sci. Rep.* 4, 4706. doi:10.1038/srep04706.

Zheng, Y., Chen, J., Craven, M., Choi, N. W., Totorica, S., Diaz-Santana, A., Kermani, P., Hempstead, B., Fischbach-Teschl, C., López, J. A., et al. (2012). In vitro microvessels for the study of angiogenesis and thrombosis. *Proc. Natl. Acad. Sci. U. S. A.* 109, 9342–7. doi:10.1073/pnas.1201240109.
